# Standardization of Epidemiological Surveillance of Acute Poststreptococcal Glomerulonephritis^[Author-notes ofac346-FM1]^

**DOI:** 10.1093/ofid/ofac346

**Published:** 2022-09-15

**Authors:** Kate M Miller, Chris Van Beneden, Malcolm McDonald, Thel K Hla, William Wong, Helen Pedgrift, David C Kaslow, Thomas Cherian, Jonathan R Carapetis, Amy Scheel, Anna Seale, Asha C Bowen, Hannah C Moore, Theresa Lamagni, Bernardo Rodriguez-Iturbe

**Affiliations:** Wesfarmers Centre of Vaccines and Infectious Diseases, Telethon Kids Institute, University of Western Australia, Nedlands, Western Australia, Australia; CDC Foundation, Atlanta, Georgia, USA; James Cook University, Cairns, Queensland, Australia; Wesfarmers Centre of Vaccines and Infectious Diseases, Telethon Kids Institute, University of Western Australia, Nedlands, Western Australia, Australia; University of Western Australia, Perth, Western Australia, Australia; Fiona Stanley Hospital, Murdoch, Western Australia, Australia; Starship Children’s Hospital, Auckland, New Zealand; Queensland Health, Cairns, Queensland, Australia; PATH, Seattle, Washington, USA; MMGH Consulting GmBH, Geneva, Switzerland; Wesfarmers Centre of Vaccines and Infectious Diseases, Telethon Kids Institute, University of Western Australia, Nedlands, Western Australia, Australia; Perth Children’s Hospital, Nedlands, Western Australia, Australia; Emory University School of Medicine, Atlanta, Georgia, USA; London School of Hygiene & Tropical Medicine, London, United Kingdom; University of Warwick, Coventry, United Kingdom; UK Health Security Agency, London, United Kingdom; Wesfarmers Centre of Vaccines and Infectious Diseases, Telethon Kids Institute, University of Western Australia, Nedlands, Western Australia, Australia; Perth Children’s Hospital, Nedlands, Western Australia, Australia; Wesfarmers Centre of Vaccines and Infectious Diseases, Telethon Kids Institute, University of Western Australia, Nedlands, Western Australia, Australia; UK Health Security Agency, London, United Kingdom; Instituto Nacional de Ciencias Medicas y Nutrición “Salvador Zubirán”, Mexico City, Mexico

**Keywords:** epidemiology, glomerulonephritis, infectious disease, *Streptococcus pyogenes*, surveillance

## Abstract

Acute poststreptococcal glomerulonephritis (APSGN) is an immune complex-induced glomerulonephritis that develops as a sequela of streptococcal infections. This article provides guidelines for the surveillance of APSGN due to group A *Streptococcus* (Strep A). The primary objectives of APSGN surveillance are to monitor trends in age- and sex-specific incidence, describe the demographic and clinical characteristics of patients with APSGN, document accompanying risk factors, then monitor trends in frequency of complications, illness duration, hospitalization rates, and mortality.

This document provides surveillance case definitions for APSGN, including clinical and subclinical APSGN based on clinical and laboratory evidence. It also details case classifications that can be used to differentiate between confirmed and probable cases, and it discusses the current investigations used to provide evidence of antecedent Strep A infection.

The type of surveillance recommended depends on the burden of APSGN in the community and the objectives of surveillance. Strategies for minimal surveillance and enhanced surveillance of APSGN are provided. Furthermore, a discussion covers the surveillance population and additional APSGN-specific surveillance considerations such as contact testing, active follow up of cases and contacts, frequency of reporting, surveillance visits, period of surveillance, and community engagement. Finally, the document presents core data elements to be collected on case report forms, along with guidance for documenting the course and severity of APSGN.

## DISEASE CHARACTERISTICS

Acute poststreptococcal glomerulonephritis (APSGN) is a delayed immune-mediated sequela of infections, usually pharyngitis or skin infections, caused by nephritogenic strains of *Streptococcus pyogenes* (group A *Streptococcus* [Strep A]), or less frequently Lancefield group C or G *Streptococcus* [1]. The reported incidence of APSGN has decreased significantly worldwide. More than 95% of the APSGN disease burden occurs in low- and middle-income countries (LMICs) [2]. Although APSGN occurs infrequently in the general population of high-income countries (HICs), it continues to be common among marginalized or impoverished populations within HICs. Globally, APSGN has been estimated to account for 5000 annual deaths in the acute phase or from resultant chronic kidney disease. However, the overall contribution to chronic kidney disease is poorly characterized and may be substantial in populations where there is both a high incidence of APSGN, especially subclinical disease, and high prevalence of chronic kidney disease with etiologies other than APSGN such as diabetes myelitis [3].

Acute poststreptococcal glomerulonephritis occurs most commonly in children aged between 5 and 12 years; in HICs, it increasingly affects adults older than 60 years, especially those with chronic medical conditions [4]. The incidence of clinical APSGN in males is approximately twice that of females; however, the incidence of subclinical APSGN is similar in both sexes [5]. The epidemiological characteristics of APSGN, such as age groups most at risk and seasonal trends in incidence, generally reflect those of the preceding Strep A pharyngitis or Strep A impetigo [6]. Acute poststreptococcal glomerulonephritis signs and symptoms occur after a 1- to 2-week latent period after Strep A pharyngitis and 3–5 weeks after Strep A impetigo [7]. Epidemics or clusters of APSGN in families or communities have occurred as a consequence of both pharyngitis and impetigo [8, 9]. Epidemic APSGN mostly occur in LMICs, low-resource settings, and within Indigenous communities of HICs [4, 9], related to the emergence of new strains of highly transmissible nephritogenic Strep A, usually found in impetigo lesions.

Acute poststreptococcal glomerulonephritis may be asymptomatic (subclinical), characterized by microscopic hematuria and reduction in serum complement, or symptomatic (clinical) with acute nephritic syndrome and occasionally with nephrotic syndrome [10]. In prospective studies of families with an index case of APSGN, subclinical APSGN has been found to occur 4 times more frequently than symptomatic cases [11]. Clinical APSGN diagnosis is based on clinical features and laboratory evidence. Typical clinical features include hematuria, edema, hypertension, and oliguria. Proteinuria up to a nephrotic range, complaints of lethargy, generalized weakness, or anorexia can also occur. Laboratory findings include microscopic hematuria with or without proteinuria, hypocomplementemia, in which the level of one or more components of complement are reduced, evidence of antecedent Strep A infection, and sometimes elevated serum creatinine. The circulating level of complement component C3 is almost universally low in the acute phase of APSGN. In the first week of APSGN, a small number of patients also have low C4 [12]. Acute poststreptococcal glomerulonephritis is usually benign and self-limiting, although <1% of cases experience acute kidney injury with crescent formation (extracapillary proliferation) on kidney biopsy [13]. Management of the clinical features remains critical, as high-quality medical care improves outcomes, particularly among children [14]. Treatment of APSGN focuses on managing hypertension and edema [15]. Penicillin can be administered if the Strep A infection is still present at the time of presentation to eradicate the nephritogenic strain [1]. Use of penicillin to treat children with impetigo who are also household contacts of patients with APSGN may prevent new APSGN cases [16].

## OBJECTIVES OF SURVEILLANCE FOR ACUTE POSTSTREPTOCOCCAL GLOMERULONEPHRITIS

An effective routine surveillance system for APSGN serves to monitor trends in the following (1) age- and sex-specific distribution of clinical and subclinical APSGN; (2) demographic and clinical characteristics of people with confirmed clinical and subclinical APSGN; (3) disease burden estimates; and (4) cases of APSGN over time and space, to detect clusters or outbreaks.

Enhanced surveillance structures are ideal adjuncts to routine surveillance but are not required in every surveillance system. Enhanced surveillance may also aim to determine and monitor the following: (1) age- and sex-specific incidence of clinical and subclinical APSGN; (2) the clinical outcomes and the frequency of complications of APSGN, such as acute kidney injury, hypertension, recurrent or chronic proteinuria, and chronic kidney disease; (3) the proportion of confirmed APSGN cases secondary to different antecedents, e.g., Strep A pharyngitis, impetigo, and/or scabies; and (4) the environmental, behavioral, and/or clinical risk factors associated with clinical and subclinical APSGN.

Additional surveillance objectives may be addressed by specialized surveillance programs, vaccine trials, or research projects. These additional activities, conducted outside routine surveillance programs, can help describe selected genotypic or phenotypic features of Strep A isolates (strains) causing APSGN, such as *emm* types, presence of vaccine antigens, and antimicrobial susceptibility. Specialized surveillance could potentially: (1) record strain-specific disease burden; (2) identify strain-specific outbreaks; (3) predict or evaluate the effectiveness of prospective or existing vaccines; (4) monitor temporal trends in Strep A strains causing APSGN; and (5) track over time antimicrobial resistance among Strep A isolates preceding APSGN.

## CASE DEFINITIONS AND CASE CLASSIFICATION

Standardized case definitions are important for obtaining accurate surveillance data, enabling comparisons of surveillance data across jurisdictions, and monitoring the impact of interventions. The definitions and methods presented here may also be used as clinical endpoints for vaccine efficacy trials and for postlicensure effectiveness studies.

The laboratory tests required to confirm a case of APSGN may not be available at all surveillance sites or may be unavailable at the time for a variety of reasons. Therefore, APSGN cases will be considered definite, if laboratory confirmation is available; or probable, if laboratory confirmation is not available or is limited. Table 1 provides proposed case definitions and classifications of APSGN for the purpose of surveillance.

**Table 1.** Case Definitions and Classification of APSGN for Surveillance

Clinical APSGN	Confirmed clinical (symptomatic) APSGN	Requires clinical AND laboratory evidence:
		Clinical evidence requires at least 2 of the following findings:
		Macroscopic hematuria: visible blood in the urine causing it to be pink, red, brownish-red, or tea-colored. Edema: any evidence of definite facial puffiness, pitting peripheral edema, ascites, or other clear evidence of generalized edema. Hypertension: Stage 1 or Stage 2 using age, sex, and height appropriate cut offs [17]
		Laboratory evidence requires all 3 of the following findings:
		Microscopic hematuria: >10 red blood cells/mm^3^ on urine microscopy; Reduced serum C3 level: tested within 4 weeks of symptom onset; and Evidence of antecedent Strep A infection: elevated or rising ASO or ADB titers^a^ OR isolation of Strep A from throat or skin sore culture OR positive RADT or NAAT from throat swab.
	Probable clinical APSGN	Requires some clinical evidence of symptomatic glomerulonephritis but does not meet ALL required clinical and laboratory evidence of confirmed clinical APSGN.
		Probable clinical APSGN requires meeting 1 of the following combinations of clinical and laboratory evidence of glomerulonephritis:
		1 clinical evidence criteria AND at least 2 laboratory evidence criteria OR 2 clinical evidence criteria AND at least 1 laboratory evidence criteria OR 3 clinical evidence criteria AND no laboratory evidence criteria
Subclinical APSGN	Confirmed subclinical (or asymptomatic) APSGN	Requires the presence of ALL 3 of the following findings:
		Microscopic hematuria: >10 red blood cells/mm^3^ on urine microscopy; Reduced serum C3 level; and Evidence of antecedent Strep A infection: elevated or rising ASO or ADB titers OR isolation of Strep A from throat or skin sore culture OR positive RADT or NAAT from throat swab
	Probable subclinical APSGN	Requires both laboratory and epidemiologic evidence of APSGN (epidemiologic evidence is needed in the absence of evidence of antecedent Strep A infection).
		Laboratory evidence of subclinical APSGN requires both of the following findings:
		Microscopic hematuria: >10 red blood cells/mm^3^ on urine microscopy; and Reduced serum C3 level
		Epidemiologic evidence includes the following:
		Glomerulonephritis occurs in a person who has close contact with a person with definite or probable clinical APSGN or lives in the same discrete community or village

Abbreviations: ADB, anti-deoxyribonuclease B; APSGN, acute poststreptococcal glomerulonephritis; ASO, antistreptolysin O; NAAT, nucleic acid amplification test; RADT, rapid antigen detection test; Strep A, group A streptococcal.

NOTE: Probable subclinical APSGN: Because abnormal urinary sediment (urinary tract infections) can be common in some communities with high rates of APSGN, and in these communities, background antistreptococcal antibody titers are often elevated, all subclinical cases require evidence of low C3. Elevated antistreptococcal antibody titers in the absence of C3 level testing is insufficient to qualify as a probable subclinical case.

a Interpretation of Strep A serology results can be difficult in communities with high incidence or prevalence of skin or upper respiratory Strep A infections. See Supplementary Appendix 2 for guidance.

### Other Definitions

#### Close Contacts

Close contacts are individuals who had stayed overnight in the house of a confirmed case in the 2 weeks preceding the onset of their illness.

#### Outbreak Surveillance

An outbreak of APSGN is defined as 2 or more probable or confirmed cases in persons from the same community with onset within 1 week of each other and with at least 1 case in a person with a reduced complement C3 or C4. Alternatively, an outbreak of APSGN can also be defined as at least 1 confirmed case and 2 probable cases in persons from the same community, with illness onset within 1 month of each other and the affected persons are not known contacts of each other [4, 18].

## SPECIMEN COLLECTION AND DETECTION OF GROUP A *STREPTOCOCCUS*

Clinical specimens and laboratory diagnostics required for the diagnosis and classification of APSGN cases are summarized in Table 2 below and includes evidence of both nephritis and preceding Strep A infection.

**Table 2.** Specimen Collection and Laboratory Testing for APSGN

Specimen	Microbiological Test	Results Indicating APSGN
Urine	Urine appearance	Macroscopic hematuria: blood visible in the urine causing it to be pink, red, brownish-red or tea-colored
	Urine microscopy	Microscopic hematuria: RBC count >10/mm^3^
		RBC appearance: deformed, acanthocytes, or red cell casts
Blood	Serum complement levels: C3	Low C3 levels, as indicated by local laboratory criteria
	ASO, ADB titers	Increased or rising titers^a^
Throat swab	Throat culture	Growth of *Streptococcus pyogenes*^b^
	Rapid test	Positive RADT or NAAT^a^
Swab of active impetigo lesion	Skin culture	Growth of *Streptococcus pyogenes*^b^

Abbreviations: ADB, anti-deoxyribonuclease B; APSGN, acute poststreptococcal glomerulonephritis; ASO, antistreptolysin O; NAAT, nucleic acid amplification test; RADT, rapid antigen detection test; RBC, red blood cell; Strep A, group A streptococcal.

a Further detail on serology is provided in Supplementary Appendix 2.

b APSGN has also been associated with *Streptococcus zooepidemicus*. Other streptococcal strains such as group C/G *Streptococcus* may be possible causes of APSGN.

### Evidence of Preceding Streptococcal Infection

Nephritis may be associated with multiple clinical syndromes, including both infectious and noninfectious etiologies (see Differential Diagnosis in Supplementary Appendix 1). Therefore, evidence of a preceding Strep A infection is a required part of the case definition for APSGN. Evidence of preceding infection includes the following: (1) increased or rising antistreptolysin O (ASO) or anti-deoxyribonuclease B (ADB) titers; (2) positive throat culture for Strep A; (3) positive nucleic acid amplification test assay for Strep A from a throat swab; (4) positive rapid Strep A carbohydrate antigen test on a throat swab in a child whose clinical presentation suggests a high pretest probability of Strep A pharyngitis; and/or (5) Strep A isolated from skin culture on at least 1 active impetigo lesion [19]. See Supplementary Appendix 2 for further detail regarding evaluation of preceding Strep A infection.

### Serum Complement Assays

Serum complement level is a blood test that can be used to measure the presence of complement proteins C3 or C4; these are typically depressed in response to Strep A due to activation of complement pathways. Although a small proportion of cases with APSGN has been reported to have normal C3 levels [20], most studies that clearly document the post-Strep A etiology of nephritis have found transient hypocomplementemia to be present in all, or almost all, cases [21]. Low C4 levels are present in a small number of APSGN cases, although this is rarely present in association with normal C3 [12]. It should be noted that complement levels usually remain reduced for the first 4 weeks, but they normalize within 8 weeks in 97% of patients and within 12 weeks in 100% of patients [21]. Therefore, a normal C3 that is tested >4 weeks after symptom onset cannot be used to exclude APSGN. Because there are other causes of low C3 levels in nephritis, such as C3 glomerulopathy and systemic lupus erythematosus, all cases of APSGN should have normalization of their C3 level 8–12 weeks after symptom onset.

### Urine Microscopy

This test is used to identify the number of red cells present in the urine. Elevated urinary red cell count occurs in APSGN with reasonable sensitivity and specificity [22]. The red cell count must be >10/mm^3^ to fulfill the microscopic hematuria criteria for APSGN diagnosis. The red cells characteristically have the appearance of a glomerular origin with deformed red blood cells, acanthocytes, and red cell casts, although not all laboratories report these specific results.

### Kidney Biopsy

Kidney biopsy is not required for the diagnosis of APSGN for the purpose of surveillance and is rarely clinically indicated, particularly among children. Kidney biopsies are occasionally done to observe disease progression, usually in adults. Biopsy can also be helpful when there are unusual clinical features such as nephrotic syndrome, rapidly progressive disease, and persistently low serum complement that raise diagnostic uncertainties. In APSGN, kidney biopsy characteristically shows diffuse proliferative glomerulonephritis with subepithelial electron-dense deposits [23].

## CASE ASCERTAINMENT AND SURVEILLANCE SETTINGS

In clinical practice, only a small proportion of people with APSGN will present with clinical features that require medical treatment. As such, only a minority of people with clinical APSGN are detected [24]. However, for most surveillance systems, APSGN surveillance will focus on ascertainment of clinical cases. Data on subclinical cases may be sought in enhanced surveillance programs during outbreaks, epidemics, or through contact testing of clinical cases.

APSGN is a notifiable condition in some countries and regions, where probable or confirmed cases are required by law to be immediately reported to local or state health authorities. Because clinically notified diseases depend on clinician knowledge, healthcare providers should be engaged with, and regularly be reminded of, their responsibility to report cases. Other common data sources used for APSGN surveillance include hospital records covering admission logs and discharge diagnosis, primary care records from outpatient and emergency departments, laboratory databases, or health insurance databases. Considerations for using administrative health databases to identify cases are provided in Supplementary Appendix 3.

People with more severe APSGN will often present directly to the hospital or possibly via a community health service. Because severe APSGN can require hospitalization and renal replacement therapy, hospital- and laboratory-based surveillance of streptococcal serology, coupled with coded diagnosis of acute renal injury or renal replacement therapy, can provide an adjunct to surveillance programs. In LMICs or subnational regions, especially in rural and remote settings, surveillance should be expanded to include community-level care through general practitioners, nurses, trained health workers, and community health centers where resources allow. Hospital- and laboratory-based surveillance should be sufficient in HICs or in regions where patients are routinely referred to hospitals for specialist care by a pediatrician and/or kidney physician (nephrologist). Case ascertainment may be active or passive (see Supplementary Appendix 4).

For each data source, surveillance staff should adhere to the following: (1) know the purpose of the data source, e.g., whether data have been routinely collected as part of patient care, mandatory collection of data under legal mandates, collected for research purposes, or other; (2) identify any legal mandates governing the operations of the data source that may affect the accessibility or quality of data from that source; and (3) describe the representative population for the data.

## TYPES OF SURVEILLANCE

The selection of surveillance strategies depends on specific epidemiologic and clinical characteristics of the disease outcome of interest, the overall surveillance objectives, surveillance location, services’ accessibility, and the resources available (see Supplementary Appendix 5 for surveillance definitions). Because APSGN is rare in most HICs, surveillance for APSGN is expected be limited to LMICs or communities with marginalized or impoverished populations within HICs where the disease remains common. The minimal and enhanced surveillance strategies for APSGN are described in Table 3.

**Table 3.** Surveillance Strategies for APSGN

*Minimal surveillance*
Minimal surveillance for clinical APSGN is facility-based, passive surveillance. Passive surveillance is based on clinical signs, symptoms, and a diagnosis recorded in health facility databases and microbiological data from laboratory databases. Minimal surveillance may be adequate if routine medical care of patients with suspected APSGN includes urine microscopy, C3 complement assay, ASO, and ADB titers, and swab of skin lesions (if present) or of the pharynx (if a sore throat or signs of pharyngitis are present) and the laboratory meets quality standards. Reporting sources, which include microbiologists, laboratory scientists, clinicians, and infection control practitioners, can be instructed to report all APSGN cases to the surveillance team. Standard case report forms may be provided to the health facilities or laboratories to encourage completion and submission to the surveillance program.
*Enhanced surveillance*
Enhanced surveillance of syndromic clinical APSGN is prospective, active, facility-based surveillance. Information is obtained from review of hospital records for each APSGN case, including outcomes, laboratory results, and clinical history. Additional information collected is used to confirm a clinical and/or laboratory diagnosis. Active surveillance maximizes case ascertainment and data collection through review of a line listing of potential cases from clinical and laboratory reports from emergency department databases, hospital admission or discharge log databases, outpatient clinics, or laboratories. Where hospital and microbiological data are computerized, surveillance personnel routinely obtain electronic line listings of all probable cases and positive throat or skin culture or serologic tests. Where data are not computerized, surveillance staff regularly liaise with hospital medical staff in pediatrics, nephrology, and intensive care and routinely review the relevant and available laboratory results to identify any new patients with APSGN [25, 26]. Asymptomatic contacts of people with APSGN are actively investigated to detect potential subclinical cases of APSGN. Some programs may have experts in medical diagnosis, usually experienced clinicians, to confirm, qualify, and assess the available diagnostic information and the accuracy of the disease code. A key component of enhanced surveillance is regular feedback of data/information to healthcare workers and others involved in the surveillance process. This critical communication should engage community healthcare workers in the process so that regular feedback informs their clinical practice. Enhanced surveillance that captures all or most APSGN cases in a clearly defined population under surveillance, and for which accurate census numbers for the population under surveillance are available, can be used to calculate APSGN incidence.
*Specialized surveillance systems or research studies*
• Specialized surveillance includes the collection of patient specimens (eg, throat or skin cultures) during the initial Strep A infection that precedes the onset of APSGN, and enables characterization (eg, *emm* type, antimicrobial resistance profiles) of the Strep A strains causing the APSGN.

Abbreviations: ADB, anti-deoxyribonuclease B; APSGN, acute poststreptococcal glomerulonephritis; ASO, antistreptolysin O.

NOTE: A quality management plan should be written before the start of surveillance to establish and ensure the quality of processes, data, and documentation associated with surveillance activities. Furthermore, all surveillance should be conducted in accordance with ethical guidelines (see Supplementary Appendix 6).

## SURVEILLANCE POPULATION

A surveillance protocol should clearly describe enrollment eligibility criteria. Most protocols are expected to survey children between less than 15 years old; however, age eligibility may vary between sites, depending on local needs and capacity. Persons with underlying immunocompromising conditions or chronic diseases, and pregnant or lactating women should not be excluded from surveillance.

The surveillance population includes all eligible at-risk people in the household and community where cases of APSGN are identified. This population, or denominator, must be properly characterized *a priori* to derive meaningful disease burden estimates. Without an accurate account of all people in the population who could potentially be evaluated for APSGN, disease estimates may be under- or overestimated [27, 28].

Because APSGN is a relatively rare disease in most populations, it is preferable to conduct surveillance across a large population to maximize the number of cases ascertained. This minimizes the confidence intervals around the point estimate of disease incidence. However, large populations are usually not well defined through demographic surveillance. Data accuracy must be assured if government-derived census data are used to determine the community’s demographic profile, such as the number of people in relevant age categories. Ongoing demographic surveillance might be necessary to generate reliable burden estimates if surveillance extends over a long period of time or if population is not stable because of mobility or other logistic factors. This is a particular challenge (1) if the population is covered by numerous hospitals or (2) if there is a substantial likelihood that cases occurring within the surveillance region may attend a tertiary or specialist hospital outside the surveillance region. Cases occurring in people residing outside the defined catchment area should be excluded. One alternative is to conduct surveillance in a smaller, high-risk population over a longer period of time, thus increasing the number of person-years at risk.

Healthcare utilization surveys can be used to determine those accessing healthcare to estimate the population served, or the denominator, that corresponds to the cases of interest [29, 30]. This may assist when surveillance is based in a sentinel hospital or where select primary care facilities serve a portion of a population residing in the geographical catchment. The denominator is the number of patients within the geographical catchment area who would be expected to attend that primary care facility if signs and symptoms of APSGN develop.

Considerations in choosing a population include likely incidence of APSGN, representativeness of the wider population when considering generalizability, accuracy of total and age-subgroup population data, and ease of case ascertainment. This includes the (1) number and accessibility of surveillance sites/hospitals and (2) likelihood that cases will attend these hospitals. Additional factors include availability of trained staff to conduct surveillance, quality of record keeping in hospitals, or potential to improve this, and availability and quality of relevant laboratory investigations. Availability of kidney biopsy is also a factor.

## SPECIAL CONSIDERATIONS FOR ACUTE POSTSTREPTOCOCCAL GLOMERULONEPHRITIS SURVEILLANCE

### *International Classification of Diseases* Diagnosis Codes

There is no specific code for APSGN; therefore, if using the *International Classification of Diseases* (ICD) codes, then all codes that include the term glomerulonephritis, hematuria, proteinuria, or nephritic/nephrotic syndrome and acute kidney injury should be cross-checked to validate actual cases (see Supplementary Appendix 7 for a full list of ICD codes). If surveillance relies on ICD diagnosis codes, an additional Strep A-specific extension code will be required to classify the confirmed case of APSGN by Strep Lancefield group, that is, XN6LP for Strep A (ICD version 11). Note that a diagnosis code assigned to a patient may be based on a clinical assumption of a specific Strep A infection without being confirmed by laboratory testing. Acute poststreptococcal glomerulonephritis may also be caused by Lancefield group C/G *Streptococcus*, as recently reported during an epidemic of APSGN [12]. Understanding local practices in coding is essential to interpreting and using ICD codes. Care should be taken to note any subtle differences between the international ICD versions used, as ICD diagnosis codes may differ.

### Active Follow-Up of Cases

Active follow-up of APSGN cases is not required for routine surveillance. However, enhanced surveillance programs may follow persons with confirmed or probable APSGN for 3 to 12 months to identify and document resolution of symptoms or signs, including hypertension, edema, hematuria, proteinuria, and hypocomplementemia. It is important to check for any persistent sequelae of APSGN such as sustained hypertension, chronic kidney disease, or persistent proteinuria. The number and causes of severe outcomes and death should be collected. Children usually recover uneventfully from APSGN, although nephrotic syndrome and, rarely, progressive azotemia may develop. Adults have a much higher incidence of azotemia and complications that include heart failure. The mortality may be as high as 20% and is age dependent. Although the long-term prognosis of APSGN is usually regarded as good, there may be a greater risk of chronic kidney disease in patients with a “second hit” of kidney damage, mostly due to diabetes mellitus. The follow-up frequency is dependent on initial severity of acute kidney injury. Those having the mildest disease require infrequent follow-up, whereas those with severe disease (∼5%) require more frequent and longer term follow-up to determine the risk of chronic kidney disease. The typical case may be followed annually; however, the total duration and extent of follow-up of the illness outcome of patients with APSGN will be determined by the specific protocol and available resources.

### Active Follow Up of Contacts

If a case of APSGN is detected, household contacts may be prospectively evaluated because of the high familial incidence [11]. Familial incidence is due to household proximity and acquisition of the same nephritogenic Strep A bacteria rather than because of a familial, genetic trait. Investigation should include asymptomatic contacts of index cases of APSGN [11]. Both adult and child contacts of confirmed cases are identified and assessed by community health nurses or experienced health workers. Contacts are examined for the presence of skin sores, scabies, facial and peripheral edema, and hematuria; their blood pressure is measured and recorded. Note that this is usually only done for research purposes, or in response to a community outbreak of APSGN, to determine the size of the outbreak and/or to identify people for targeted interventions, mainly antibiotic treatment. However, most public health authorities instead choose to use outbreak definitions based on the number of clinical cases of APSGN in a defined population and then to conduct interventions without screening. For example, in the event of any outbreak of APSGN in an Aboriginal community in the Northern Territory of Australia, all household members of APSGN patients, and all children with skin sores, receive a single dose of benzathine benzylpenicillin. It is not currently known how subclinical APSGN translates to long-term kidney outcomes, so screening for asymptomatic cases will remain, in most instances, a research tool.

### Frequency

The schedule of passive surveillance will be dictated by the pattern of case presentation at the surveillance site(s). Case ascertainment can be improved if the active surveillance includes regular visits to surveillance sites to review clinical and laboratory records for missed cases and to share with healthcare workers the goals and methods of Strep A surveillance. This may be informed by the average duration of hospitalization for inpatients or timing of follow-up visits for outpatients at the surveillance facilities.

### Period of Surveillance

Given the low incidence of APSGN relative to Strep A pharyngitis and impetigo, longer periods of surveillance are generally required to obtain accurate estimates of incidence and of strain distribution. Several years of surveillance are generally required to evaluate temporal trends, or identify epidemics, which tend to be cyclical (occurring approximately every 5 years) [9].

### Season

Acute poststreptococcal glomerulonephritis incidence is seasonal in many places, with peaks generally correlated with antecedent oropharyngeal and skin infections [9]. If possible, surveillance staff should conduct surveillance across all seasons to capture the changes in disease prevalence over time. In areas where seasonality of pharyngitis and skin infections are well described in the surveillance population, limiting surveillance to months when most cases of APSGN are likely to occur has efficiencies but will produce inflated prevalence estimates and annual incidence rates.

### Community Engagement

Community engagement helps provide a considered approach to surveillance and ensures that the project has community value. It also provides the community with an opportunity to clearly express their values and concerns and develop a degree of ownership. The time required to forge relationships between surveillance staff and communities should not be underestimated and must be built into the surveillance protocol at the outset. The level of community engagement in the design, implementation, monitoring, and evaluation of surveillance will depend on available resources and community capacity. Key stakeholders include community leaders, teachers, health staff, and volunteers.

One-off and ongoing feedback of aggregated surveillance data, and its interpretation, should be provided to the communities and regional/remote health services. Funding for activities to support the feedback process should be built into all surveillance budgets and protocols from the outset. It will often require translation to local language and then into an accessible format. Feedback is best given by local health providers; adequate support and training to local health providers should be provided as necessary.

## DATA COLLECTION AND CASE REPORT FORMS

Case report forms should be based on collecting only the information required to achieve the surveillance objectives. Supplementary Appendix 8 provides a list of recommended and optional variables for inclusion in case report forms.

General surveillance variables include unique identifier, date and time of first enrollment or specimen collection, and site where participant is seen, such as clinic, school, and household. The documentation of each encounter should also contain a surveillance visit number/episode number if repeated episodes from the same person are included. Key demographic variables include date of birth, if available, or age (in days or months, if <12 months, and otherwise in years), sex, ethnicity/race, and residential postcode, state, and country. Clinical and epidemiologic variables include APSGN diagnostic category, epidemiologic risk factors, clinical risk factors, evidence of preceding Strep A infection, indicators of severity of illness, laboratory findings, treatment, and follow-up. Although not expected as part of routine surveillance, it may be appropriate for some enhanced surveillance programs to document the course of the episode after the onset of illness. For consistency in reporting, the course of an episode should be measured in a reproducible way. The duration of an episode of APSGN can be measured by the length of hospital stay in days or duration of clinical signs/symptoms in nonhospitalized patients. Because there are no defined grades of severity of APSGN, severity of disease can be monitored by capturing the duration of hypertension, hematuria, edema, proteinuria, and any complications.

## Supplementary Data

Supplementary materials are available at *Open Forum Infectious Diseases* online. Consisting of data provided by the authors to benefit the reader, the posted materials are not copyedited and are the sole responsibility of the authors, so questions or comments should be addressed to the corresponding author.

## Supplementary Material

ofac346_Supplementary_Data
