# Supplementary material for: Standardization of Epidemiological Surveillance of Acute Poststreptococcal Glomerulonephritis
Source: Open Forum Infect Dis. 2022 Sep 15;9(Suppl 1):S57–64. doi: 10.1093/ofid/ofac346 (PMC9474944; doi:10.1093/ofid/ofac346)
Supplement: ofac346_Supplementary_Data [file ofac346_supplementary_data.docx]

**Standardization of Epidemiological Surveillance of Acute Poststreptococcal Glomerulonephritis**

Supplementary Appendices

Table of Contents

[Appendix 1: Differential Diagnosis 2](#_Toc112339848)

[Appendix 2: Considerations and Limitations to the Evaluation of Preceding Strep A Infection 3](#_Toc112339849)

[Appendix 3: Administrative Health Databases 6](#_Toc112339850)

[Appendix 4: Comparisons of Advantages and Disadvantages of Active and Passive Case Detection Methods 7](#_Toc112339851)

[Appendix 5: Definitions of Key Surveillance Terms 8](#_Toc112339852)

[Appendix 6: Good Practice and Ethical Considerations 9](#_Toc112339853)

[Appendix 7: Acute Post Streptococcal Glomerulonephritis: ICD-11 Codes That can be Used to Identify Potential Cases of APSGN 10](#_Toc112339854)

[Appendix 8: Variables for Inclusion in Acute Post Streptococcal Glomerulonephritis Surveillance Datasets 11](#_Toc112339855)

[References 14](#_Toc112339856)

## Appendix 1: Differential Diagnosis

Occasionally, other causes of nephritis, such as systemic lupus erythematosus (SLE) may cause a similar clinical presentation to APSGN and be associated with low serum complement levels. In APSGN, the C3 should return to normal within 4-8 weeks after presentation. Therefore, C3 should always be repeated 8 weeks or more after presentation. If it remains depressed, alternative diagnoses, including C3 glomerulopathy should be considered, even when evidence of preceding streptococcal infection was present at initial diagnosis.

The differential diagnosis of patients presenting with nephritic syndrome includes other post-infectious glomerulonephritide, IgA or IgM nephropathy, SLE, infective endocarditis, Henoch- Schönlein purpura with nephritis, anti-glomerular basement membrane (GBM) disease, and granulomatosis with polyangiitis (GPA). Some of these can be diagnosed without a renal biopsy. For example, infective endocarditis may be confirmed with cardiac examination, echocardiography, and blood cultures, and SLE may be confirmed with autoimmune tests such as the anti-DNA antibody. However most require renal biopsy to determine severity and to guide therapy with involvement of a specialist such as a nephrologists or rheumatologist.

Other causes of edema, including hypoproteinemia and cardiac failure, can usually be excluded early in the clinical course. It is rare for these conditions to be confused with APSGN after an initial period of observation and some baseline investigations.

## Appendix 2: Considerations and Limitations to the Evaluation of Preceding Strep A Infection

| **Gold Standard Approach** | **Alternative Approach** | **Limitations** |
| --- | --- | --- |
| **Elevated ASO or ADB Titer(s)** | |  |
| - If patient presents within two weeks of illness, collect lab values on more than one occasion separated by 4-6 weeks to demonstrate rising titers: a four-fold or more increase in titer from acute to convalescence - *ASO titers typically peak 3-5 weeks after infection; if presentation is >3 weeks, obtain ASO titer at presentation. Repeating for a second time is of little utility - Develop age-stratified ULN values for serum ASO and ADB titers in a subset of individuals without a recent streptococcal infection in the surveillance population of interest | - A single value above the upper limit of normal (ULN) is considered sufficient for a preceding Strep A infection if not feasible to obtain multiple specimens. This is the most common approach, especially because the first sample is often delayed due to late presentation, or the latent period between infection and onset of APSGN may mean that antibody titers have already begun to rise at the time of presentation - Use of recommended ASO and ADB ULN titers when local derivations are not available (recommended upper limits are provided below) | - Development of local population ULN is not always possible due to logistics, costs, or surveillance being conducted in areas with high prevalence of streptococcal infections and thus difficulty finding children without recent infection |
| **Positive Throat Culture for Group A β-hemolytic Streptococci** | | |
| - Specimens should be obtained from the surface of both tonsils and the posterior pharyngeal wall - The sample should be cultured on a sheep-blood agar plate and incubated at 37°C for 18–24 h before reading. If no growth occurs, incubate for an additional 24 h before concluding that the culture is negative | - NAAT assays have been studied extensively and are highly sensitive for detecting Strep A.^1^ - NAAT assays offer a quick and reliable alternative to bacterial culture | - False negatives may occur if child is treated with antibiotics prior to culture collection - Strep A can exist in the carrier state; a positive throat culture does not necessarily confirm active infection - Patients with APSGN typically present weeks after initial Strep A infection |
| **Positive Rapid Diagnostic Test** | | |
| - Specimens should be obtained from the surface of both tonsils and the posterior pharyngeal wall - Use NAAT due to higher sensitivity than traditional RADT | - Use of RADT when NAAT test is not available - Validation of RADT to be used in population with culture confirmation prior to use when possible | - Strep A can exist in the carrier state; a positive throat culture does not necessarily confirm active infection - Patients with APGSN typically present weeks after initial Strep A infection - NAAT equipment is costly and does not differentiate between viable and nonviable bacteria in the specimen - RADT tests have varying sensitivities that do not differentiate between viable and nonviable bacteria, and negative results require culture |
| **Group A β-hemolytic streptococci from Active Impetigo Lesion** | | |
| - Perform full body skin exam to locate most active lesions - Sample active lesion, selecting the most purulent lesion | - Sample crusted lesions if no purulent lesions present | - Crusted lesions are more likely to become co-infected with S *aureus* or not grow on culture |

Abbreviations: ASO; anti-streptolysin O, ADB; anti-DNase B, NAAT; nucleic amplification tests, RADT; rapid antigen detection test

Strep A serology

Interpretation of Strep A serology results can be difficult in communities with high incidence or prevalence of skin or upper respiratory Strep A infections^2^. In these settings, a negative antibody test helps exclude a recent infection, but a positive test does not necessarily indicate an infection in the past few weeks. Wherever possible, titers should be interpreted by comparing acute and convalescent samples and demonstrating a titer rise between these two time-points. A four-fold increase is considered definitive, while two-fold is within the error range of serial two-fold dilutions. Convalescent samples should align with the peak antibody titers (ASO and ADB) to optimize sensitivity. The timing of the rise for ASO and ADB differ slightly at 3–5 weeks and 6–8 weeks, respectively, although this can vary between individuals. It is recommended that convalescent samples be taken from 4–6 weeks following symptom onset to best capture the rise in both titers.

An upper limit of normal (ULN) cut-off (80^th^ percentile) can be used in place of the gold standard four-fold rise in titer when paired sera are not available. Ideally, age stratified ULN values for serum ASO and ADB titers will be available for a subset of healthy individuals, without recent Strep A infection, and drawn from the surveillance population of interest. Local data, where available, should be used to determine threshold titers as values can differ between and within countries based on variables such as ethnicity, geography, and socioeconomic status. However, developing local population ULN values is not always possible due to logistics, cost, or surveillance occurring in regions where streptococcal infections are endemic, and it is difficult to identify local children without a recent infection.

Recommended upper limits of normal for anti-streptolysin O and anti- DNase B titers, in the absence of appropriate local population data.

| **Age group (years)** | **Upper limit of normal (international units/mL)** | |
| --- | --- | --- |
|  | **ASO titer** | **Anti-DNase B titer** |
| 2-4 | 160 | 240 |
| 5-9 | 240 | 320-640 |
| 10-12 | 320 | 480-640 |
| >12 | 400 | 200 |

From: Kaplan EL et al, Pediatrics 1998; 101: 86-8; Gray GC et al. J Clin Epidemiol 1993; 46: 1181-5; and Karmarkar MG et al, Indian J Med Res. 2004;119 Suppl:26-8.

Upper limit of normal (80th centile) values for serum Streptococcal antibody titers in children and adults in tropical settings where Strep A is endemic^3^

| Age Group (years) | Upper Limit of Normal (international units/mL) | |
| --- | --- | --- |
|  | ASO Titer | ADB Titer |
| 1–4 | 170 | 366 |
| 5–14 | 276 | 499 |
| 15–24 | 238 | 473 |
| 25–34 | 177 | 390 |
| >35 | 127 | 265 |

## Appendix 3: Administrative Health Databases

Administrative data from laboratory datasets and electronic medical records (EMRs) from primary healthcare and emergency departments covering whole communities can provide a timely and cost-effective surveillance option.

An important consideration when using EMRs to calculate disease estimates in a population is that the data are collected and coded as part of service delivery rather than for surveillance purposes. As such, EMRs are often prone to missing data on key fields and require the conversion of unstructured/narrative text, which can be resource-intensive and subjective. For EMRs that include or rely on free text, new methods in machine learning or deep learning could improve case identification.^4,5^Data are limited to patients who attend health services, and a subject to variance in physician’s propensity to seek microbiological confirmation, which may be subject to bias (e.g., more severe infections, more clinically ambiguous, one not responding to treatment) and underestimate disease incidence. However, an advantage to administrative data is that, in well-established systems, data are collected systematically, well-structured and are often population-based. EMRs can form the basis of enhanced surveillance by using an additional data collection form to augment routinely collected data.

Routinely collected clinic data may be insufficient for evaluating potential cases against the full criteria required to meet surveillance case definitions, especially when microbiological testing is not routinely conducted or recorded. Further, the data may be insufficient for addressing other surveillance objectives, such as variant typing and antimicrobial susceptibility testing.

## Appendix 4: Comparisons of Advantages and Disadvantages of Active and Passive Case Detection Methods

| **Advantages** | **Disadvantages** |
| --- | --- |
| ***Active surveillance*** |  |
| - Sensitive system that facilitates early detection of new cases. Early detection allows surveillance to contribute to the prevention of post infectious sequalae - Higher case ascertainment rate - More accurate identification of cases - Ability to verify information in the case of missing data or suspected data entry errors - Data collected can be comprehensive and specific to the surveillance objectives - Can evaluate the quality and effectiveness of case-finding process, thus minimizing selection bias - Allows real-time analysis and ability to respond/modify approach to surveillance and care - Can promote disease awareness and good health practices | - Can be costly and resource-intensive - Requires dedicated surveillance staff and/or extensive training and upskilling - Can be demanding on surveillance sites - Barriers to accessing communities (i.e., distance/cultural barriers) |
| ***Passive surveillance*** |  |
| - Can be conducted retrospectively - Requires fewer resources than active surveillance - Inexpensive and can cover large areas | - Responsibility for reporting new cases lies with healthcare workers/laboratory staff; thus, it can be difficult to ensure compliance by healthcare providers and other reporters - Difficulties caused by lack of standardization in terms of case definitions and coding - Tends to under-report disease - Commonly associated with incompleteness of data recording or of microbiological studies - Often difficult to confirm data recording or entry errors retrospectively |

## Appendix 5: Definitions of Key Surveillance Terms

| **Syndromic surveillance** | Syndromic surveillance refers to the use of a clinical syndrome – a constellation of symptoms and signs – as the case definition for detection of suscept cases. Syndromic surveillance can be used for initial case detection, but laboratory confirmation should occur to increase the accuracy of the system.^10^ |
| --- | --- |
| **Active surveillance** | Active case detection means that designated public health surveillance staff are directly involved in detecting cases.^10^ |
| **Passive surveillance** | Passive case detection means that health facility staff detect and report cases to the public health system.^10^ |
| **Facility-based surveillance** | Facility-based surveillance is based on ascertainment of cases in persons who seek care at health facilities, including outpatient clinics, doctors’ offices, hospitals and emergency departments.^10^ |
| **Sentinel-site surveillance** | Sentinel-site surveillance refers to a system that captures cases at one or more specialized sites, such as hospitals, clinics, schools or pharmacies.^10^ |
| **Community-based surveillance** | Community-based surveillance is the systematic detection and reporting of events of public health significance within a community-by-community members. Community-based surveillance enables earlier detection of the disease of interest and captures illnesses in persons who do not seek care in a hospital.^11^ |
| **Population-based surveillance** | Population-based surveillance attempts to capture all cases in a well-defined catchment population (for example, the entire population of a country). |
| **Healthcare utilization surveys** | Healthcare utilization surveys seek to characterize the health care-seeking behavior of ill persons by describing where ill persons sought health care for their illnesses, and soliciting reasons for not seeking health care.^12^ |
| **Unique identifier** | Unique identifiers are unique numbers or numbers and letter combinations that are allocated to a specific individual person. |

## Appendix 6: Good Practice and Ethical Considerations

**Monitoring/Audit**

A systematic and independent audit of surveillance systems should be undertaken to ensure that surveillance and surveillance-related activities were conducted following the relevant surveillance protocol, SOPs, ethical guidelines, and regulatory requirement(s) established by local public health. Existing surveillance review tools can be modified to guide the investigation (e.g., WHO’s ‘[Tools for a surveillance review: Vaccine Preventable Diseases Surveillance Standards](https://www.who.int/publications/m/item/vaccine-preventable-diseases-surveillance-standards-annex1)’). Surveillance as part of a clinical study should adhere to the ICH Guidelines for Good Clinical Practice ^9^.

**Quality Control and Quality Assurance**

A quality management plan should be written before the start of surveillance to establish and ensure the quality of processes, data, and documentation associated with surveillance activities. It encompasses both quality control (QC) and quality assurance (QA) activities.

Surveillance systems should develop SOPs to ensure confidentiality for all cases, ensure that clinical specimens and bacterial isolates obtained are not compromised by human and processing errors, validate data integrity, and maintain multiple layers of security. A SOP will ideally detail:

- Data storage. Including participants’ unique surveillance ID numbers in each respective dataset enables linkage to other datasets, such as hospital admissions, facilitating the capture of complications and ensuring that all personal identifying information is removed from research/surveillance datasets.
- Data evaluation for protocol compliance and source document accuracy.
- Document review (e.g., specimen tracking logs, questionnaires), who is responsible, and frequency.
- Who the responsible person is for addressing QA issues (correcting procedures that do not comply with the surveillance protocol) and QC issues (correcting errors in data entry).
- Staff training activities and processes for documenting surveillance staff training.
- Maintenance and strict adherence to surveillance delegation log (list of staff involved in the surveillance and their duties/roles).
- Clinical and laboratory SOPs and accreditation.
- Regular audits of surveillance data to ensure accuracy and completion.
- System for periodic and refresher training for surveillance team.

**Ethics of Surveillance**

The global network of WHO Collaborating Centres for Bioethics in collaboration with the U.S. Centers for Disease Control and Prevention developed ethical guidelines for public health surveillance, including common good, respect for persons, and good governance. The guidelines cover the (i) broad responsibility for undertaking surveillance and subjecting it to ethical scrutiny; (ii) obligation for ensuring appropriate protection and rights; (iii) considerations in making decisions about how to communicate and share surveillance data. The guidelines are available at <https://apps.who.int>. Countries should implement these guidelines and monitor them regularly. As appropriate, surveillance protocols should adhere to existing country-specific ethical guidelines.

## Appendix 7: Acute Post Streptococcal Glomerulonephritis: ICD-11 Codes That can be Used to Identify Potential Cases of APSGN

| **Clinical diagnosis** | **Associated** **ICD-11 Codes*** |
| --- | --- |
| Nephritic syndrome | GB40 |
| Nephrotic syndrome | GB41 |
| Macro haematuria | MF50.40 |
| Micro haematuria | MF50.41 |
| Haematuria unspecified | MF50.4Z |
| Proteinuria | MF96 |
| Other specified amyloidosis | 5D00.Y |
| Other specified secondary gout | FA25.1Y |
| Other specified clinical findings in specimens from the urinary system | MF8Y |
| Chronic kidney disease | GB61.0-GB61.Z |
| Pauci-immune proliferative glomerulonephritis | MF84 |
| Other specified systemic lupus erythematosus | 4A40.0Y |
| Acute Kidney Failure | GB60.0-GB60.Z |

*ICD diagnosis codes (International Classification of Disease Codes) is a medical classification list by the World Health Organization providing codes for diseases, signs and symptoms, abnormal findings, and external causes of injury or diseases ^10^.

Note there is no specific code for APSGN. Codes provided in the above table are those relating to glomerulonephritis, haematuria, proteinuria or nephritic/nephrotic syndrome that can identify potential cases for further investigation to validate actual cases.

ICD11 Codes listed in Appendix are current as of February 2022 and should be used as a guide only. Jurisdictions should be aware of country-specific modifications. Retrospective studies will need to identify ICD-10 (or ICD-9) codes used during the surveillance period.

If surveillance relies on ICD diagnosis codes, a Strep A-specific code is required to classify the infection as caused Strep A. For example, for the ICD version 11 the extension code would be XN6LP for Strep A.

## Appendix 8: Variables for Inclusion in Acute Post Streptococcal Glomerulonephritis Surveillance Datasets

| **Category of variables** | **Required variables** | **Optional variables** |
| --- | --- | --- |
| General | - Unique ID number - Date of diagnosis | - Date of enrolment |
| Demographics | - Age (in months if <1 year; years) - Sex | - Race/ethnicity - Residential address |
| Recent Strep A infection | - Yes/No | - Group A Strep pharyngitis in prior 7 days - Confirmed Group A Strep impetigo in past 30 days |
| Evidence of preceding Strep A infection | - Laboratory confirmation (Y/N) | - Diagnostic test (throat swab, NAATs, RADT, skin swab) - Diagnostic result - ASO titer (date taken and titer, date and titer if repeated) - Anti-DNase B titer (date taken and titer, date and titer if repeated) |
| Diagnostic category | - Confirmed clinical APSGN - Probable clinical APSGN - Confirmed subclinical APSGN - Probable subclinical APSGN |  |
| Diagnosis of APSGN | - Hypertension (Y/N) - Hematuria (Macroscopic / Microscopic / No) - Low C3 (Y/N) | *Clinical findings*   - Maximum blood pressure (Systolic/Diastolic) - Edema (Y/N)   - Specify: facial puffiness, pitting peripheral edema, ascites, other clear evidence of edema   *Laboratory findings*   - Urine red cell count   - Date taken - C3 level   - Date taken - C4 level   - Date taken |
| Other clinical information  (check all that apply) |  | - Proteinuria >2+ on dipstick (Y/N) - Anuria (no urine for 24 hours) (Y/N) - Acute Kidney Injury (Stage 1,2,3) ^11^ - Highest blood urea (Date and level) - Highest blood creatinine (Date and level) - Dialysis (Date) - Renal biopsy   - If yes, date and result: - Anti-nuclear antibody   - If yes, date and result: - Known contact of APSGN case (Y/N) - Reason for consultation (fever, abdominal symptoms, direct symptoms of APSGN, complications of APSGN) - Patient historical data |
| Epidemiologic features |  | - Exposure history to pharyngitis among family or household members, classmates, or other close contacts of the child - Underlying skin infections (e.g., scabies, eczema, insect bites, trauma, herpetic outbreaks, other) - Exposure history to impetigo among family or household members, classmates, or other close contacts of the child - Number of household members - Number of bedrooms in household - Household health hardware - Access to fresh running water |
| Treatment | - Hospitalized - Date of admission | *Pre-hospitalization:*   - Antibiotics prior to admission: yes/no; if yes, β-lactam (penicillins, cephalosporins), macrolides, clindamycin, tetracycline, other   *During hospitalization:*   - Date of admission - Date of discharge - Admitted to ICU - Length of stay - Antibiotics (route of administration; name of antibiotic; duration; dose) - β-lactam alone, β-lactam + clindamycin, β-lactam + other, non- β-lactam |
| Follow up confirmation of Dx |  | - APSGN diagnosis remains unchanged (Date of last follow-up) - C3 level re-tested and normal (Date of most recent testing and level) |
| Long term adverse events |  | - Stages of Chronic Kidney Disease (1-5) - Hypertension - Persistent proteinuria. |
| Microbiology | - Participant unique ID number - Name of reporting laboratory - Specimen collection date - Specimen unique ID - Type of test - Anatomic site sampled - Specimen type - Strep A (or S pyogenes) identified: yes/no | - β-hemolytic Streptococcus identified: yes/no   - If yes, group identified (choose one): A, B, C, G or other - Anti-streptolysin O (ASO) and anti-DNAase B antibodies - Storage/transport identification number - Place/site of transfer of isolate for additional testing - Further testing ordered (e.g., emm typing, whole genome sequencing, anti-streptococcal antibody titers, speciation of large-colony β-hemolytic Streptococcus, antibiogram, etc.) |

## References

1. Parker KG, Gandra S, Matushek S, Beavis KG, Tesic V, Charnot-Katsikas A. Comparison of 3 Nucleic Acid Amplification Tests and a Rapid Antigen Test with Culture for the Detection of Group A Streptococci from Throat Swabs. *Journal Applied Laboratory Medicine.* 2019;4(2):164-169.

2. Okello E, Ndagire E, Muhamed B, et al. Incidence of acute rheumatic fever in northern and western Uganda: a prospective, population-based study. *The Lancet Global Health.* 2021;9(10):e1423-e1430.

3. Steer AC, Vidmar S, Ritika R, et al. Normal ranges of streptococcal antibody titers are similar whether streptococci are endemic to the setting or not. *Clinical Vaccine Immunology.* 2009;16(2):172-175.

4. Solares JRA, Raimondi FED, Zhu Y, et al. Deep learning for electronic health records: A comparative review of multiple deep neural architectures. *Journal of Biomedical Informatics.* 2020;101:103337.

5. Wang Z, Shah AD, Tate AR, Denaxas S, Shawe-Taylor J, Hemingway H. Extracting diagnoses and investigation results from unstructured text in electronic health records by semi-supervised machine learning. *PLoS One.* 2012;7(1):e30412.

6. World Health Organization. Surveillance standards for vaccine-preventable diseases. 2018.

7. World Health Organization. A definition for community-based surveillance and a way forward: results of the WHO global technical meeting, France, 26 to 28 June 2018. *Eurosurveillance.* 2019;24(2).

8. Deutscher M, Van Beneden C, Burton D, et al. Putting surveillance data into context: the role of health care utilization surveys in understanding population burden of pneumonia in developing countries. *Journal of Epidemiology and Global Health.* 2012;2(2):73-81.

9. U.S. Department of Health and Human Services. *E6(R2) Good Clinical Practice: Integrated Addendum to ICH E6(R1) Guidance for Industry* Maryland2018.

10. World Health Organization. *The International Statistical Classification of Diseases and Health Related Problems ICD-10: Tenth Revision. Volume 1: Tabular List.* Vol 1: World Health Organization; 2004.

11. Kellum JA, Lameire N, Aspelin P, et al. Kidney disease: improving global outcomes (KDIGO) acute kidney injury work group. KDIGO clinical practice guideline for acute kidney injury. *Kidney International Supplements.* 2012;2(1):1-138.
